# Effect of the Compounding Conditions of Polyamide 6, Carbon Fiber, and Al_2_O_3_ on the Mechanical and Thermal Properties of the Composite Polymer

**DOI:** 10.3390/ma12183047

**Published:** 2019-09-19

**Authors:** Young Shin Kim, Jae Kyung Kim, Euy Sik Jeon

**Affiliations:** 1 Industrial Technology Research Institute, Kongju National University, Cheonan-daero, Seobuk-gu, Cheonan-si 31080, Chungcheongnam-do, Korea; people9318@gmail.com; 2 Department of Mechanical Engineering, Graduate School, Kongju National University, Cheonan-daero, Seobuk-gu, Cheonan-si 31080, Chungcheongnam-do, Korea; kjk8431@gmail.com

**Keywords:** composite material, extrusion, injection molding, mechanical properties, thermal properties, Morphology, melt flow rate.

## Abstract

Among the composite manufacturing methods, injection molding has higher time efficiency and improved processability. The production of composites via injection molding requires a pre-process to mix and pelletize the matrix polymer and reinforcement material. Herein, we studied the effect of extrusion process conditions for making pellets on the mechanical and thermal properties provided by injection molding. Polyamide 6 (PA6) was used as the base, and composites were produced by blending carbon fibers and Al_2_O_3_ as the filler. To determine the optimum blending ratio, the mechanical properties, thermal conductivity, and melt flow index (MI) were measured at various blending ratios. With this optimum blending ratio, pellets were produced by changing the temperature and RPM conditions, which are major process variables during compounding. Samples were fabricated by applying the same injection conditions, and the mechanical strength, MI values, and thermal properties were measured. The mechanical strength increased slightly as the temperature and RPM increased, and the MI and thermal conductivity also increased. The results of this study can be used as a basis for specifying the conditions of the mixing and compounding process such that the desired mechanical and thermal properties are obtained.

## 1. Introduction

Composites refer to materials produced by combining two or more materials to complement the shortcomings of each material while taking advantage of the features of each material [1,2]. Composites have been applied in aerospace applications in the past; since then, they have also been applied in various other industries [3]. The expanding areas across which composites are being applied have encouraged many studies, especially on polymer composites strengthened using polymers, as the matrix in combination with various fibers to enhance their stiffness of the polymers. 

Polyamide 6 (PA6) [4], also known as nylon 6, is synthesized by the ring-opening polymerization of caprolactam, which contains six carbon atoms in its cyclic structure. Thus, each repeating unit of the polymer consists of six carbon atoms, hence the name poly(hexano-6-lactam) or simply polyamide 6. Among all amides, PA6 is commercially the most important. Owing to the polarity of the amide group and the strong attraction between chains, PA6 has advantages such as superior toughness, impact strength, tensile strength, abrasion resistance, and chemical resistance [5]. Various fiber fillers have been used to improve the properties of the polymer matrix. As a representative example, carbon fibers (CFs) are attractive engineering materials, primarily because they have a high strength-to-weight ratio. In addition, their stiffness and strength properties with respect to weight (specific stiffness and specific strength, respectively) are superior to those of a range of popular engineering materials including polymers [6,7,8]. Another material that is used to reinforce the polymer matrix is alumina (Al_2_O_3_), which is a ceramic material with high thermal conductivity and is used to enhance the low thermal conductivity of the polymer matrix. Alumina can also be applied to improve the heat dissipation performance of home appliances because of its electrical insulation properties [9]. 

Various engineering methods are employed in the process of manufacturing composites consisting of a polymer matrix and fiber reinforcements. Autoclaving, the method mainly applied in the aerospace field, can be used to manufacture high-quality reproducible parts, including those requiring high fiber volume fractions and low porosity [10]. Even though this method can produce excellent composites, many out-of-autoclave (OoA) processes have been developed to avoid the high manufacturing cost and time-consuming manufacturing process associated with autoclaves [11]. In particular, resin transfer molding (RTM) is increasingly being used to manufacture large-sized structures consisting of composites, such as fuselage frames, turbo-propeller blades, boats, and wind turbine blades [12,13]. 

Among these composite manufacturing methods, injection molding has lower cost, higher time efficiency, and improved processability. Moreover, injection molding is among the manufacturing methods that can be used to mass-produce polymer composites [14]. Hence, many studies have been conducted on the production of composites using injection molding. Zhang et al. [15] analyzed the relationship between the screw structure and properties of recycled glass fiber-reinforced flame-retardant nylon 46 (RGFFRPA46) in injection molding. Ahmed et al. [16] demonstrated the correlation between extruder configuration and fiber aspect ratio, and hence the overall strength of the composite. Yahui et al. [17] fabricated composites via injection molding according to the variation in the initial length of the CF and analyzed the physical and thermal properties. However, they reported that although the initial fiber length had no effect, the process conditions played a significant role. The production of composites via injection molding requires a pre-process to mix and pelletize the matrix polymer and reinforcement material. During extrusion, the matrix (fed through the main feeder) and fibers (fed through the side feeder) are blended and pelletized to obtain composite pellets, wherein the fibers are randomly distributed and made short as a result of extensive fiber breakage during extrusion. 

In the present study, to analyze the effect of the extrusion process conditions on the mechanical and thermal conductivities, pellets were prepared using filler in the polymer matrix, and various mixing ratios and extrusion tests were performed. With PA6 as the base, composites were produced by blending CFs, which improve the strength, and Al_2_O_3_ as the filler, which increases the thermal conductivity. The mechanical and thermal properties of the composite were then analyzed according to the blending ratio and compounding process conditions. In addition, the morphology was analyzed by Scanning Electron Microscope (SEM) to determine the dispersibility of the fillers according to the process conditions.

## 2. Experimental Details

### 2.1. Materials

PA6 with Hyosung 1011br, RV 2.4 (Korea) was used as the matrix resin in this work. In addition, spherical alumina (Al_2_O_3_) was used as the thermally conductive filler and particle reinforcement (Dongkuk R&S Co. Ltd., Gimhae, Korea). The chemical purity of Al_2_O_3_ exceeded 99.9%, and the DSP-AS series with 245 free Na+ [ppm] and 11 free Cl− [ppm] was used. Alumina with spherical particles of various sizes was used to improve the thermal conductivity by increasing the packing density [18,19]. Spherical alumina particles with a mean diameter of 5, 10, and 25 [μm] were selected. The particles were mixed in the ratio of 10:1:1. [20,21]. The CF (tensile strength ≥5000 MPa; elastic modulus, 250 GPa; density 1.8 kg/m^3^; diameter, 7 μm; length, 3–12 mm) was supplied by HYOSUNG Co. Ltd. (Korea). Table 1 lists the blending ratios of PA6, Al_2_O_3_, and CF. The mechanical properties of these samples with different blending ratios were analyzed.

### 2.2. Preparation of Composites

The samples to be tested were prepared in two steps. First, the mixture was pelletized using the same compounding conditions at the blending ratios provided in Table 1. The samples whose mechanical properties were analyzed were fabricated by injection molding. During the compounding of each sample in Table 1, an initial rotational screw speed of 300 RPM and melting temperature of 250 °C were used. In the second step, the pellets were fabricated by changing the conditions of the compounding process to determine the composition. Injection molding was performed with an injection temperature of 260 °C and injection pressure of 6.5 MPa. 

When polyamides are exposed to humidity, the absorption of moisture affects some mechanical properties of PA6 compounds. In particular, the moisture acts as a plasticizer. This implies that properties like strength, stiffness, elongation, and toughness are affected. The plasticizing action of moisture is manifested in an increase in the impact strength and toughness of polyamide. Due to the polymeric chain distribution in the resin, the material experiences a reduction of strength and stiffness and an increase of elongation. [22,23]

Therefore, drying of PA6 is essential, and was performed in this study. The PA6 used in this study was applied on samples whose moisture content was controlled below 0.12% on average. Additionally, before blending the PA6 filler, the fibers were dried at 85 °C in a hopper dryer for 4 h to remove moisture. Fiber-reinforced thermally conductive PA6 composites were manufactured by a TSE 32 twin-screw extrude machine (UNEEPLUS Co. Ltd., Hwaseong, Korea) at a screw speed of 300~500 RPM and melting temperature range of 250~290 °C. The screw diameter in the extrusion machine was 32 mm, and the length-diameter ratio was 40. Samples were fabricated by applying NE-80 (i.e., a clamping force of 80 tons) (Woojin Plaimm Co. Ltd., Boeun, Korea) for injection molding. The effect of compounding on the properties of the sample was analyzed by fixing the injection molding temperature at 260 °C and injection pressure at 65 MPa.

### 2.3. Characterization

Tensile strength (Ts) tests were carried out at room temperature according to ASTM D638-14 [24] using a computer controlled QM 100TM universal testing machine ((Qmesys Co. Ltd., Anyang-si, Gyeonggi-do, Korea) with a cross-head speed of 5 mm/min. Flexural tests were also conducted on the universal testing machine at room temperature according to ASTM D790-17 [25]. The notched impact strengths of the composites were determined with a QM700A IZOD Type impact tester (Qmesys Co. Ltd., Anyang-si, Gyeonggi-do, Korea) at room temperature according to ASTM D256-10 [26]. Each test was repeated three times to obtain each reported value. 

In addition, the melt flow index (MI) was measured via a method that involved determining the rate of extrusion of molten thermoplastic resins using an extrusion plastometer [27]. After a specified preheating time, the resin was extruded through a die with a specified length and orifice diameter under the prescribed conditions of temperature, load, and piston position in the barrel. The MI was measured after drying the sample at 100 °C for 2 h, using a QM280A (Qmesys Co. Ltd., Anyang-si, Gyeonggi-do, Korea) at a temperature of 235 °C and load of 1.0 kg according to ASTM D1238. [27]

The melting and crystallization behaviors of the composites were determined using a differential scanning calorimeter. Differential scanning calorimetry (DSC) measurements were performed under nitrogen atmosphere using a DSC Q20 V24.11 Build 124, calibrated with high purity standards (indium), (TA Instruments, Lukens Drive, New Castle, USA) in the range of 50–250 °C. The samples used for DSC assessment, approximately 5–6 mg, were taken from the subsurface of the compounded specimens. The samples were heated to 250 °C at a heating rate of 10 °C/min, and then cooled to 50 °C at 10 °C /min. The crystallization and second melting curves were recorded. [28,29] The crystallization and melting enthalpies were deduced by running a curve integral. The degree of crystallinity (Xc) was determined from DSC scans using the following equation

(1) $$X_{c}=\frac{\Delta \mathrm{Hm}}{\left(1-\varphi_{1}-\varphi_{2}\right)\times \Delta H_{m}^{0}}\times 100\%$$

where ΔHm is melting enthalpy of the samples, $\Delta H_{m}^{0}$ is the enthalpy value of melting of the 100 % crystalline form of PA6 (240 J/g) [17,30], $\varphi_{1}$ is the mass fraction of Al_2_O_3_ and $\varphi_{2}$ is the mass fraction of CF.

The thermal conductivity (Tc) of the composites was determined by the NETZSCH Geraetebau GmbH (LFA447), using a xenon flash lamp as the IR source. The thermal diffusivity was measured using the flash method, which is a noncontact measurement method, with no contact resistance with the sample, and the thermal conductivity was tested according to ASTM E1461. The testing samples had a diameter and a thickness of 12.7 mm and 2 mm, respectively. [31]

The morphology of the composites was observed by SEM (Sigma 500, Carl-Zeiss, Jena, Germany) using an acceleration voltage of 10 kV. The samples were cryogenically fractured in liquid nitrogen, and all the fractured surfaces were coated with platinum to enhance the image resolution and prevent electrostatic charging. [27,32]

## 3. Results and Discussion

### 3.1. Properties of the PA6/Al_2_O_3_/CF Composites

Table 2 lists the mechanical properties (tensile strength, flexural strength, impact strength) and melt flow rates according to the blending ratio of PA6/Al_2_O_3_/CF. The tensile strength decreased as the proportion of Al_2_O_3_ increased, which was added to improve the thermal conductivity. The elongation decreased with increasing proportion of Al_2_O_3_ and CF. The addition of a rigid filler or fiber restricts the chain mobility of polymer molecules; this may lead to the formation of micro-cracks in the composites. Furthermore, increased stress concentration at the ends of fibers is another reason for crack formation in the matrix. It is known that, when the extent of cracks in the specimen reaches to a critical level especially in matrix surrounding fibers, matrix cannot resist to applied load and then cracks initiate in those regions [33,34,35]. The flexural strength decreased with increasing proportion of Al_2_O_3_. The mechanical strength of the composites containing alumina was low because alumina powder has weak interfacial bonding with the polymer matrix [36,37]. In addition, the mechanical strength decreased due to void formation between PA6 and alumina [38]. The extent to which the impact strength was influenced by the blending ratio of alumina and CF was difficult to confirm. Nevertheless, the impact strength of the specimens containing alumina and CF was greater than that of PA6/Al_2_O_3_/CF(0-0) without these materials. 

MI represents the material characteristics and is indicative of the rheological behavior of polymers [39,40,41,42]. The MI decreased as the content of alumina increased. The MI of PA6/Al_2_O_3_/CF (0-0) was 63 g/10 min, while that of PA6/Al_2_O_3_/CF (65-0) decreased to 44 g/10 min. 

While the heat transfer coefficient of the polymer PA6 was 0.29, that of the specimen with alumina was over three times higher. For the specimen with 60% alumina, its heat transfer coefficient was highest at 1.13. This study found that when a composite contained alumina, its mechanical strength (tensile, flexural, and impact strengths) and MI dropped, whereas its heat transfer coefficient increased. The mechanical properties improved significantly as the proportion of alumina and CF was increased. For specimens with 5% and 10% of CF each, the three types of mechanical strengths increased proportionally with the increase in carbon content.

The flow rate also decreased as the CF content increased [42]. Furthermore, comparing PA6/Al_2_O_3_/CF (60-0) with PA6/Al_2_O_3_/CF(60-10), the difference in MI was 23.4 when the CF content increased by 10%. On the other hand, comparing PA6/Al_2_O_3_/CF (50-10) with PA6/Al_2_O_3_/CF(60-10), the MI decreased by approximately 6 when the alumina content increased by 10%.

CF had a greater effect on the MI because the MI decreased more when the CF content increased than when the alumina content increased. In addition, comparing PA6/Al_2_O_3_/CF(60-0) with PA6/Al_2_O_3_/CF(60-10), all the measured properties were relatively high except for the MI value. 

The determination of the mechanical properties and thermal conductivity of each sample according to the blending ratio enabled determination of the MI value at which the tensile strength and thermal conductivity were the highest. This is also the MI value most suitable for injection molding. Considering this, additional experiments were performed by changing the compounding conditions. To analyze the mechanical properties, thermal conductivity, crystallization and melting properties, and morphology according to the process conditions of the compounding, the blending ratio of PA6/Al_2_O_3_/CF (60-10), which exhibited the highest tensile strength and thermal conductivity, was selected based on the experimental results in Table 2.

### 3.2. Mechanical Properties and MI of PA6/Al_2_O_3_/CF (60-10) Composites under Varying Process Parameters

**Figure 1:**
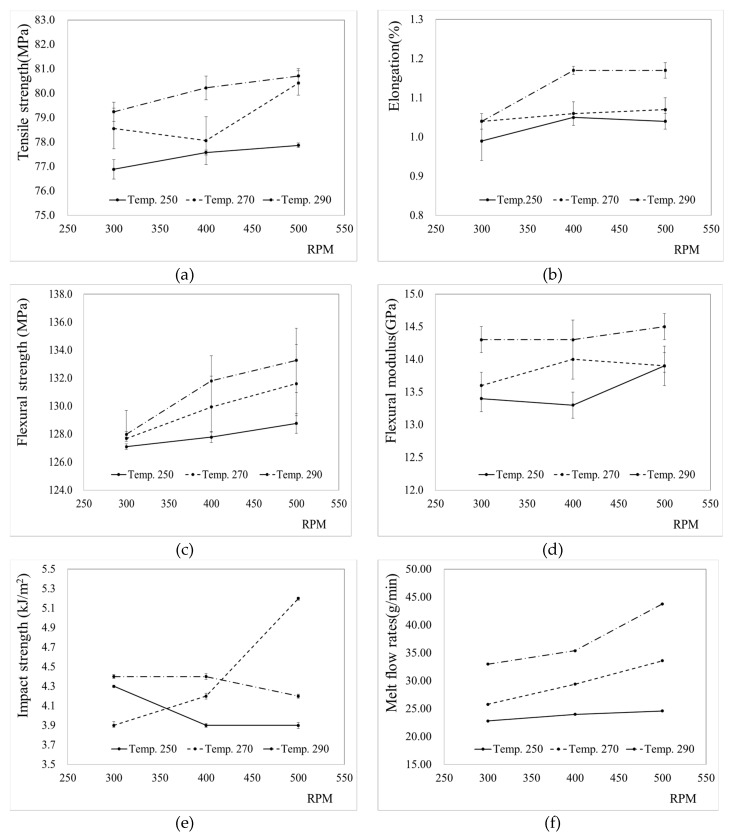
Mechanical properties and MI of PA6/Al_2_O_3_/CF (60-10) composites with varying process parameters: (**a**) tensile strength, (**b**) elongation, (**c**) flexural strength, (**d**) flexural modulus, (**e**) impact strength, and (**f**) melt flow rates.

To analyze the mechanical properties and MI, pellets were produced by changing the compounding conditions of PA6/Al_2_O_3_/CF (60-10), and samples were fabricated under a fixed condition of injection molding. Nine compounding conditions were set by varying the temperature and RPM, and the tests were performed three times for each condition. The injection molding samples were fabricated under the same conditions using the pellets produced by compounding. The parameters for injection molding were as follows: melt temperature, 260 °C; injection pressure, 65 MPa; holding pressure, 60 MPa; holding time, 10 s; cooling time, 20 s. Table 3 outlines the experimental results for each compounding condition. The tensile strength, flexural strength, impact strength, and melt flow rates were measured as a function of the temperature and RPM. The experimental results were analyzed by plotting the mechanical properties as a function of the process conditions in Figure 1 based on the data in Table 3.

Figure 1a shows the tensile strength according to the RPM and temperature. As the temperature and RPM increased, the tensile strength increased slightly. This appears to be the result of an increase in the blending state of the materials as the temperature and RPM increased. With the increase in RPM, the tensile strength increased by 1 MPa at a compounding temperature of 250 °C, while it increased by 1.8 MPa at 270 °C and by 1.5 MPa at 290 °C. On the other hand, the tensile strength increase due to the change in temperature was 2.3 MPa at 300 RPM, 2.6 MPa at 400 RPM, and 2.8 MPa at 500 RPM. This implies that temperature has a greater effect on the tensile strength than RPM. 

Figure 1b shows a graph of the elongations according to the RPM and temperature. The difference between the maximum and minimum elongation was 0.18% at the same RPM (500 RPM) as the temperature changed. When the temperature was 290 °C, the difference in elongation as a result of varying the RPM was 0.13%. This suggests that the effect of temperature on the elongation is greater than that of RPM. 

Figure 1c shows a graph of the flexural strength according to the RPM and temperature. The difference between the maximum and minimum flexural strengths when varying the RPM was 5.3 MPa at 290 °C, while that arising from varying the temperature was 4.5 MPa at 500 RPM. This suggests that the flexural strength increased as the temperature and RPM increased, and also indicated that the effect of RPM on the flexural strength was greater than that of temperature. 

Figure 1d shows the flexural moduli. With increasing temperature and RPM, the flexural modulus increased overall, except in some sections where a reverse trend was observed. The flexural modulus was found to be more affected by temperature than by RPM. 

The impact strength is plotted in Figure 1e, as a function of RPM for various temperatures; however, no significant trend was found. With increasing RPM, the impact strength at 250 °C and 290 °C decreased, while that at 270 °C increased.

Figure 1f shows the measured MI values of the pellets prepared under various extrusion conditions (ASTM D1238). In this case, the effect of temperature seemed to be greater than that of RPM. At 500 RPM, the MI showed the largest increase to 19.2 g/10 min as temperature changed from 250 °C to 290 °C. This implies that the viscosity of the mixture will be reduced with increasing RPM and temperature. Due to this, dispersibility of composite materials is assumed that. Analysis of the mechanical properties confirmed that the tensile strength, elongation, flexural strength, flexural modulus, and MI tended to increase as the RPM and temperature increased. 

### 3.3. Thermal Conductivity of the Composites

**Figure 2:**
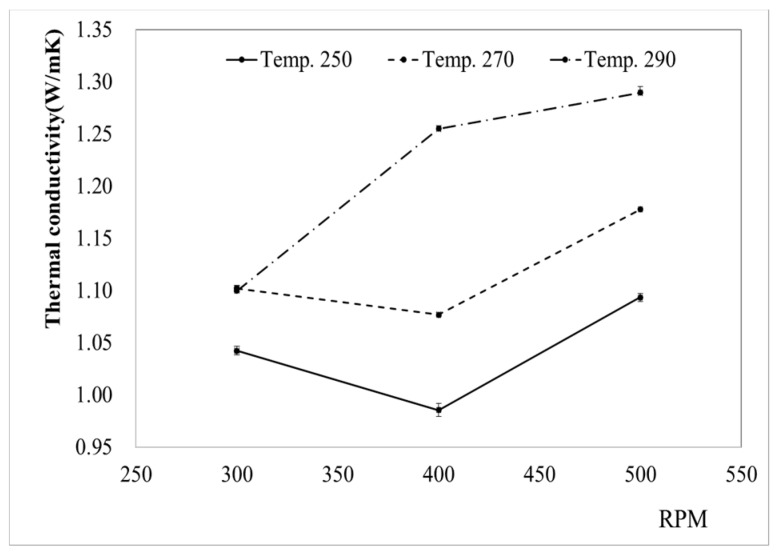
Thermal conductivity of PA6/Al_2_O_3_/CF (60-10) composites for varying process parameters.

Table 4 and Figure 2 show the results of the thermal conductivity measurements for various values of the RPM and temperature. The thermal conductivity did not show a linear relationship with RPM, but tended to increase slightly as the temperature increased. When the RPM was increased from 300 to 400, the heat transfer coefficient decreased at 250 °C and 270 °C, whereas it increased at 290 °C. When the RPM was increased to 500, the heat transfer coefficient rose at all these temperatures. The lowest thermal conductivity was 0.986 W/mK, obtained at 250 °C and 400 RPM, while the highest was 1.29 W/mK, obtained at 290 °C and 500 RPM. The heat transfer coefficient was also high when the temperature and RPM were set to the highest level. 

### 3.4. Crystallization and Melting Properties

**Figure 3:**
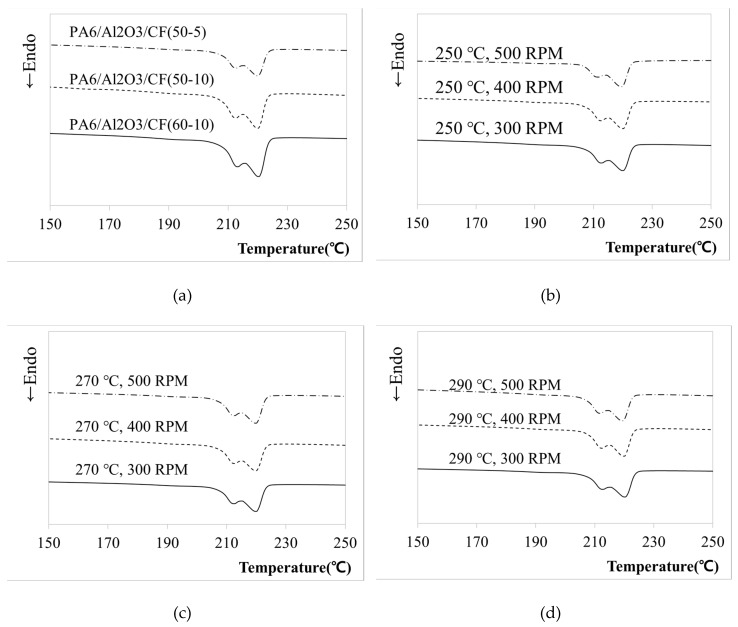
Differential scanning calorimetry (DSC) heating thermograms of PA6/Al_2_O_3_/CF composites for various conditions. (**a**) DSC thermograms of composites with different weight ratios, (**b**) Samples fabricated at 250 °C, (**c**) Samples fabricated at 270 °C, (**d**) Samples fabricated at 290 °C.

In semi-crystalline polymers, the crystallization behavior of the matrix has a significant influence on the physical properties of the corresponding composites [30,43,44]. DSC analysis was carried out to determine the crystallization and melting properties according to the change in RPM and temperature. Table 5 outlines the enthalpy changes according to the conditions. In addition, Figure 3a shows the melting curves with various compositions and Figure 3b, c and d show the DSC thermograms according to temperature and RPM during heating for each sample fabricated in this study. Semi-crystalline polymers can exhibit multiple melting endotherms which are generally attributed to the melting of imperfect crystals formed during the crystallization or different crystal types depending on the presence of specific fillers. Multiple melting behaviors have also been reported for PA6 and its composites in related studies [30,45,46,47,48]. As is well known, c-phase crystals are preferred at higher cooling rate, while the a-phase crystals dominate in slow cooling rate [30,49], and the two crystal forms are melting at about 215 °C (γ-form) and 225 °C (α-form), respectively. We can conclude that the double melting endotherms in the PA6 composite were probably caused by two different crystal structures. The peak temperatures during heating ranged from 219 °C to 221 °C. The Total melting enthalpy (ΔH) was calculated from the Tm. Degree of crystallinity (X_c_) was calculated according to Equation (1). It is easy to find that the X_c_ was increased as the fillers increased. It can be concluded that CF and Al_2_O_3_ will restrict or block the movement and arrangement of PA6 chains, leading to more imperfect crystals and lower crystallinity. The change of X_c_ value with the change of content was observed, but it was difficult to find a meaningful change of X_c_ value with the change of process conditions. 

### 3.5. Microstructures of the Composites

**Figure 4:**
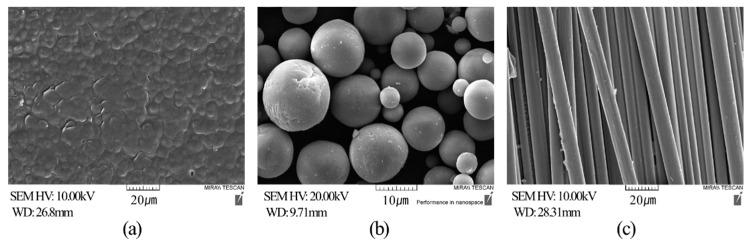
SEM images of raw materials (**a**) PA6; (**b**) Al_2_O_3_; (**c**) Carbon fiber.

**Figure 5:**
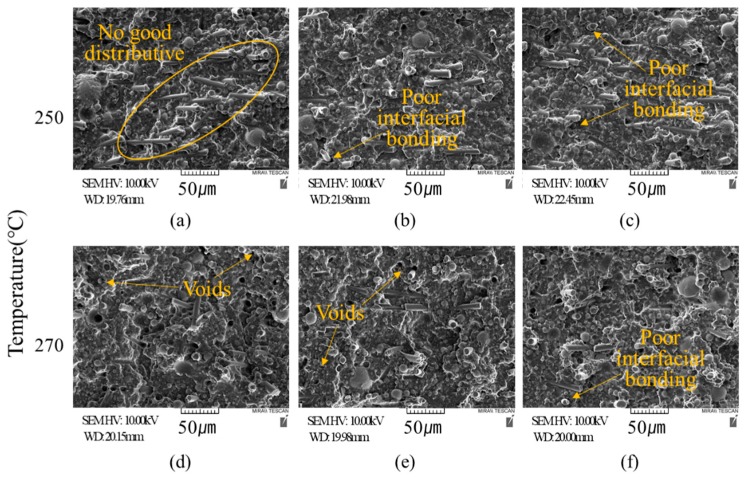

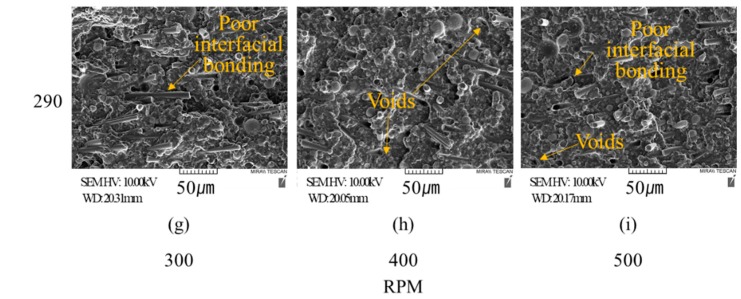
SEM images of the reinforced composites with various process parameters. (**a**) 250 °C, 300 RPM; (**b**) 250 °C, 400 RPM; (**c**) 250 °C, 500 RPM; (**d**) 270 °C, 300 RPM; (**e**) 270 °C, 400 RPM; (**f**) 270 °C, 500 RPM; (**g**) 290 °C, 300 RPM; (**h**) 290°C, 400 RPM; (**i**) 290 °C, 500 RPM.

Figure 4 shows the SEM images of the raw materials; Figure 4a shows the PA6 matrix, Figure 4b shows spherical alumina, and Figure 4c, CF. Figure 5 shows the SEM images of the cross sections of the PA6/Al2O3/CF (60-10) samples fabricated under different process conditions. The samples were cryogenically fractured for the SEM analysis. The CFs and Al2O3 are shown to be irregularly distributed in the matrix. It could be seen that the fibers aligned in different directions and were fine dispersed by shear force during extruding, which is of great importance for making CF reinforced polymer composites with mechanical properties [30]. In Figure 5a, CFs are concentrated in specific parts. This phenomenon is a typical disadvantage of composites and deteriorates the mechanical and thermal properties [50,51]. Figure 5a, c, d, and g show that the fibers and PA6 matrix are separated and are drawn out, and that they have voids. This indicates poor contact between the fibers and polymer matrix [52]. The Al_2_O_3_ particles, which were added as reinforcement to improve the thermal conductivity, must be in contact with each other to form a heat bridge and thereby improve the thermal conductivity. However, the SEM results in Figure 5 show that the Al_2_O_3_ particles are not in direct contact with each other, but are surrounded by polymer molecules, thereby preventing the thermal conductivity from improving. Hence, the ratio of reinforcement materials needs to be higher to improve the thermal conductivity of the composites. 

## 4. Conclusions 

In the present study, using pellets prepared with fillers contained in the polymer matrix, extrusion tests were carried out according to various compounding ratios and process conditions in order to analyze the effect of the extrusion process conditions on the mechanical and thermal conductivity characteristics. Alumina was effective toward improving the thermal conductivity; however, mechanical degradation occurred, whereas with CF, the mechanical properties were improved. 

When pellets were manufactured by injecting molding at elevated temperatures and RPM, some improvements were observed in the mechanical properties of the PA6 composites, and thermal conductivity was also confirmed. In addition, the MI was confirmed to be affected by the pellet production temperature. The SEM results revealed separation between the fillers and polymer matrix, and irregular distribution of the fibers. The composites exhibited voids and weak contact. Further research is needed to improve adhesion through the surface treatment of fillers. The results of this study including the mechanical, thermal, and morphological properties of the PA6 composites according to various blending ratios and process conditions could aid in the palletization of PA6 composites with the desired properties. 

## Figures and Tables

**Table 1:** Composition of samples with varying content of PA6, Al_2_O_3_, and carbon fiber used for the preparation of polymer blends.

Sample Designation	Composition (wt%)		
	PA6	Al_2_O_3_	CF
PA6/Al_2_O_3_/CF (0-0)	100		
PA6/Al_2_O_3_/CF (60-0)	40	60	-
PA6/Al_2_O_3_/CF (65-0)	35	65	-
PA6/Al_2_O_3_/CF (50-5)	45	50	5
PA6/Al_2_O_3_/CF (50-10)	40	50	10
PA6/Al_2_O_3_/CF (60-10)	30	60	10

**Table 2:** Properties of PA6/Al_2_O_3_/CF composites with different weight ratios.

Sample Designation	Tensile Strength (MPa)	Elongation (%)	Flexural Strength (MPa)	Impact Strength (kJ/m^2^)	Melt Flow Rates (g/10 min)	Thermal Conductivity (W/mK)
PA6/Al_2_O_3_/CF (0-0)	72.2 ± 0.1	30 ± 0.01	105.6 ± 0.5	2.9 ± 0.1	63.0	0.29 ± 0.002
PA6/Al_2_O_3_/CF (60-0)	60.4 ± 0.2	1.33 ± 0.01	100.7 ± 0.7	3.3 ± 0.1	46.2	1.13 ± 0.001
PA6/Al_2_O_3_/CF (65-0)	55.1 ± 0.2	1.0 ± 0.02	99.3 ± 0.6	3.1 ± 0.2	44.4	0.98 ± 0.004
PA6/Al_2_O_3_/CF (50-5)	69.5 ± 0.4	1.83 ± 0.02	115.8 ± 1.5	3.9 ± 0.3	37.2	0.70 ± 0.001
PA6/Al_2_O_3_/CF (50-10)	75.3 ± 0.3	1.15 ± 0.01	133.9 ± 0.8	4.4 ± 0.1	28.8	0.85 ± 0.001
PA6/Al_2_O_3_/CF (60-10)	76.9 ± 0.6	0.99 ± 0.01	127.4 ± 1.2	4.3 ± 0.2	22.8	1.29 ± 0.001

**Table 3:** Mechanical properties and MI of PA6/Al_2_O_3_/CF(60-10) composites for various combinations of the two processing parameters.

Parameters of Compounding Process		Tensile Strength (MPa)	Elongation (%)	Flexural Strength (MPa)	Flexural Modulus (GPa)	Impact Strength (kJ/m^2^)	Melt Flow Rates (g/10 min)
Temp. (°C)	RPM						
250	300	76.9 ± 0.4	0.99 ± 0.01	127.1 ± 2.1	13.4 ± 0.2	4.3 ± 0.01	22.8
250	400	77.6 ± 0.1	1.05 ± 0.00	127.8 ± 0.5	13.3 ± 0.1	3.9 ± 0.02	24.0
250	500	77.9 ± 0.1	1.04 ± 0.02	128.8 ± 0.7	13.9 ± 0.2	3.9 ± 0.03	24.6
270	300	78.6 ± 0.8	1.04 ± 0.02	127.7 ± 2.0	13.6 ± 0.3	3.9 ± 0.04	25.8
270	400	78.1 ± 0.9	1.06 ± 0.03	129.9 ± 2.2	14.0 ± 0.1	4.2 ± 0.03	29.4
270	500	80.4 ± 0.5	1.07 ± 0.03	131.6 ± 2.8	13.9 ± 0.2	5.2 ± 0.01	33.6
290	300	79.2 ± 0.3	1.04 ± 0.02	128.0 ± 0.2	14.3 ± 0.1	4.4 ± 0.02	33.0
290	400	80.2 ± 0.4	1.17 ± 0.01	131.8 ± 1.8	14.3 ± 0.1	4.4 ± 0.03	35.4
290	500	80.7 ± 0.2	1.17 ± 0.02	133.3 ± 2.3	14.5 ± 0.2	4.2 ± 0.02	43.8

**Table 4:** Thermal conductivity of the PA6/Al_2_O_3_/CF(60-10) composites for various process parameters of compounding.

Parameters of Compounding Process		Thermal Conductivity (W/mK)
Temp. (°C)	RPM	
250	300	1.043 ± 0.004
250	400	0.986 ± 0.006
250	500	1.094 ± 0.004
270	300	1.102 ± 0.003
270	400	1.077 ± 0.002
270	500	1.178 ± 0.001
290	300	1.100 ± 0.003
290	400	1.155 ± 0.003
290	500	1.290 ± 0.006

**Table 5:** Differential scanning calorimetry DSC results for PA6/Al_2_O_3_/CF(60-10) composites with varying weight ratios and process parameters.

Sample Designation	Process Parameters of Compounding		DSC Data		
	Temp. (°C)	RPM	T_m_ (°C)	ΔH (J/g)	X_c_ (%)
PA6/Al_2_O_3_/CF(50-5)	250	300	220.84	32.14	29.76
PA6/Al_2_O_3_/CF(50-10)			220.59	27.52	28.67
PA6/Al_2_O_3_/CF(60-10)			220.66	21.92	30.44
PA6/Al_2_O_3_/CF(60-10)	250	400	220.55	21.35	29.65
	250	500	220.14	20.22	28.08
	270	300	220.45	22.30	30.97
	270	400	220.25	22.45	31.18
	270	500	220.06	23.37	32.46
	290	300	221.24	21.92	30.44
	290	400	220.29	22.28	30.94
	290	500	219.91	22.26	30.92
